# The impact of parenting styles on undergraduate students’ emotion regulation: The mediating role of academic-social student-faculty interaction

**DOI:** 10.3389/fpsyg.2022.972006

**Published:** 2022-10-07

**Authors:** Hao Yao, Shuzhen Chen, Xiulin Gu

**Affiliations:** ^1^Faculty of Education, East China Normal University, Shanghai, China; ^2^School of Education, Soochow University, Suzhou, China

**Keywords:** emotion regulation, parenting style, student-faculty interaction, structural equation model, multiple mediating effects

## Abstract

Based on the survey data of 4,462 undergraduate students in Zhejiang Province, mainland China, this study investigated the influence of parenting styles on emotion regulation and the mediating role of student-faculty interaction. The study found that: (1) Male students scored significantly higher than female students on emotion regulation, overprotective parenting style and student-faculty interaction. (2) Parenting style has a direct positive effect on emotion regulation, and warm parenting style has a much greater effect on emotion regulation than overprotective parenting style. (3) The mediating effect of student-faculty interaction in the relationship between parenting style and emotion regulation holds true, with the mediating effect of academic student-faculty interaction being much higher than that of social student-faculty interaction. (4) The influence of warm parenting style on emotion regulation relies more on the direct effect, while the influence of overprotective parenting style on emotion regulation relies more on the mediating effect of student-faculty interaction.

## Introduction

Emotion regulation is the process by which individuals use emotion regulation strategies to regulate their emotional experiences, physiological responses and behavior in order to achieve their emotion regulation goals in a given situation (Gross, 2015). Correct emotional adjustment is not only significant for individuals to maintain psychological balance, but is also fundamental to the experience of well-being (Schimmack and Derryberry, 2005). Emotion regulation is one of the key indicators of mental health (Strickhouser et al., 2017; Khodamia and Sheibanib, 2020). In addition to its important function of protecting individuals’ emotional balance, emotion regulation can also have a significant impact on the performance of cognitive activities, the establishment of harmonious interpersonal relationships and the maintenance of physical and mental health (Leersnyder et al., 2013). When individuals have difficulty in regulating their emotions, they are more likely to be stressful and suffer from psychological or mental illness (Lane and Smith, 2021).

University is an extremely challenging transitional period in an individual’s life. In addition to the pursuit of self-identity and professional career, it is a critical time for transitioning into adult roles, which results in many undergraduate students experiencing extra academic and life pressures (Puffer et al., 2021). The impact of COVID-19, in particular, makes emotion regulation particularly important for students as they face sudden changes in their studying, living environment and interpersonal relationships (Mariani et al., 2021). Post-traumatic stress disorder, eating disorders, substance dependence, social anxiety, borderline personality and depression are all associated with difficulties in emotion regulation among undergraduate students (Aurora and Klanecky, 2016; Weiss et al., 2019). Anxious students tend to use more maladaptive strategies to cope with negative life events (Arndta et al., 2013). And when they fail to adapt to them, it can lead to anxiety, depression and pessimism (Aldao and Nolen-Hoeksema, 2010). When undergraduate students face adversity or passive interpersonal relationships, if they suppress their emotions for a long time without being able to regulate them, they are prone to emotional disorders that affect their physical and mental health. In more serious cases, irreversible consequences such as suicide and anti-social behavior will be induced, while those who are able to use emotion regulation strategies flexibly and appropriately tend to have better interpersonal relationships and social adaptability (Liao et al., 2022). Thus, emotion regulation in undergraduate students is of great research importance, both clinically and in theoretical guidance.

Family and school education play an important role in the development of students’ emotion regulation (Darling and Steinberg, 1993). It is widely accepted that the development of emotion regulation in adolescents is based on positive parenting styles and school teacher-student relationships (Garaigordobil, 2020). Therefore, the main aim of this study is to examine the links between parenting styles, student-faculty interaction and emotion regulation. More specifically, we are interested in the potential mediating role of different student-faculty interaction between two types of parenting styles (warm and overprotective parenting styles) and emotion regulation.

The theoretical construction of the relationship between these variables (direct and indirect) is based on two main theories. According to Bronfenbrenner and Morris (1998) ecosystem theory, the family is an irreplaceable micro-environment that influences the mental health, personality and behavior of individuals. Parenting style is one of the core elements of the family environment. The attitude, behavior and emotions displayed by parents in the upbringing of their children have a significant impact on their children’s emotion regulation skills. From constructionist perspective (Contreras-Quiroz et al., 2022), emotion development is a state that develops dynamically through the interaction of emotional expressions. As school teachers are important persons in the social interaction of undergraduate students, individual emotions develop through constant interaction and feedback with teachers and eventually develop into specific emotional patterns. Therefore, based on these two theoretical models, we have developed a conceptual model by linking family parenting styles, student-faculty interaction and emotion regulation.

## Literature review

### Parenting styles and emotion regulation

Parenting styles play an important role in the development of emotion regulation (Efstratopoulou et al., 2022). From psychological perspective, parenting style refers to the pattern of behavior in raising and educating children, including parenting behavior, attitudes and emotional support (Şiţoiu and Pânişoară, 2022). Attachment theory suggests that parenting styles determine the child’s attachment and the emotional connection between family members. And it has a great impact on the individual’s personality and emotion regulation (Shaver and Mikulincer, 2010). According to personality traits, parenting styles are categorized as warm and overprotective. Warm parenting means that parents are able to perceive and respond to the needs of their children in a timely and sensitive manner, providing them with sufficient love, support and understanding. Overprotective parenting refers to parents’ excessive attention and control over their children’s daily behavior. The overprotection is detrimental to individual’s independence (Segrin et al., 2012; Li, 2021).

Compared to overprotective parenting style, warm parenting style is conducive to emotion regulation of undergraduate students. Firstly, the family relationship is closer under a warm parenting style so that individual’s negative emotional experiences such as loneliness and sense of insecurity are significantly reduced (Balan et al., 2017). It is also easier to build trust with others with their more evident outgoing, pleasant and emotionally stable nature (Delgado et al., 2022). With a warm parenting style, parents also tend to provide more autonomy support, which is more conducive to fostering positive emotions of children (Froiland, 2015). Autonomy support could exercise their individual autonomy (Moè, 2016). Meanwhile, when parents encourage their children to be independent and meet the need for autonomy, children are more inclined to think proactively and master strategies to cope with difficult situations (Marcone et al., 2020). This leads to a greater sense of self-efficacy, which in turn leads to a better emotion regulation in the face of complex tasks (Salavera et al., 2022). Parents do not blame their children even when they fail and do not break the family atmosphere where children are supported and respected (Moè et al., 2020). Secondly, parents who adopt a warm parenting style tend to be empathetic and it can implicitly help their children learn to put themselves in others shoes (Wang et al., 2021). It also allows children to form a good social network with the outside world and learn emotional skills in the external environment (Van Der Kaap-Deeder et al., 2019). In contrast, an overprotective parenting style prevents student from developing independence and autonomy as they grow up. Because it reduces their exposure to difficult situations, which prevents them from acquiring effective coping strategies, and is therefore not conducive to high level of emotion regulation (Leung, 2021). When parents show excessive concern for students’ life and safety, which is out of responsibility, it may reduce students’ resilience (Kim, 2019). Petegem et al. (2020) has found that overprotective parenting style could pose risks to young people’s emotion regulation, such as social anxiety and other negative psychological emotions that could not be dissipated. Even, some overprotective parenting behavior when blocked may trigger a more controlling parenting stance toward the children (Mabbe et al., 2018), which can obviously be depressing for children.

### Parenting styles, student-faculty interaction and emotion regulation

Student-faculty interaction refers to the information exchange activities between university teachers and students within or outside the classroom. Isohätälä et al. (2019) classified student-faculty interactions as academic and social student-faculty interaction. The former is mainly about academic communication between teachers and students, while the latter is about non-disciplinary knowledge such as career planning and values outside the classroom.

Firstly, positive and effective parenting style may have a positive impact on student-faculty interaction. In the case of warm parenting styles, Stright et al. (2008) believed that children had a good teacher-faculty relationship with their teachers when their mothers adopt a warm parenting style. This may be due to the fact that a warm parenting style facilitates the acquisition of interpersonal skills and appropriate interventions, which help students learn more about social rules. It increases their subjective willingness and effectiveness in interacting with teachers (Romm and Metzger, 2021). However, the current research is not conclusive as far as overprotective parenting is concerned. Ye et al. (2022) found that overprotective parenting style did not adversely affect students’ engagement in student-faculty interaction, but affected students’ psychological well-being.

Secondly, the teacher-student relationship is an important part of students’ interpersonal relationships at school that affects the cultivation of students’ emotion regulation (Pottie and Ingram, 2008). Positive teacher-student relationships are characterized by warmth, emotional support and trust, whereas negative teacher-student relationships tend to alienate students and make them feel less supported (Maulana et al., 2014). Interaction with teachers is an important source of social support for undergraduate students. The emotional and social support provided by such a network of people profoundly affect an individual’s emotional control (Gavriluţã et al., 2022). In the process of student-faculty interaction, the teacher’s emotional support provides a pathway for individuals to share their emotions, seek understanding or vent bad feelings, thus enhancing emotion regulation (Ivcevic and Eggers, 2021). Waber et al. (2021) pointed out that in-class student-faculty interaction had a positive impact on students’ academic performance, sense of belonging, career experience, self-perception, social meaning and interaction skills, and that out-of-class student-faculty interaction promoted students’ emotion management and emotional perceptions.

Finally, student-faculty interaction may play a mediating role in the mechanism by which parenting styles influence emotion regulation. In other words, parenting styles further act on the cultivation of emotion regulation by influencing the interpersonal relationships that undergraduate students perceived in school. Social capital theory suggests that intra-family social capital like reciprocity and trust between family members is the main way to translate family capital into human capital (cognitive skills, academic achievement, etc.). That is to say, social capital outside the family is an important channel for developing non-cognitive skills such as emotion regulation (Wei, 2012). The social network between parents and teachers forms a closed interpersonal circle that provides undergraduate students with three resources: expectations, information channels and social norms, which are important mechanisms for influencing adolescents’ ability to regulate their emotional experiences and control their emotional expression (Horner and Wallace, 2013). Students with a warm parenting style have a good sense of trust in interpersonal relationships at school (Wang et al., 2021). They are driven to interact more comfortably with teachers, both academically and non-academically. The guidance and feedback they received motivate them to achieve goals and place them in a harmonious context to cultivate emotion regulation (Saariaho et al., 2018). Students with overprotective parenting styles have a low level of academic adjustment and sense of belonging to the university when they are freshmen. They are prone to problems such as psychological anxiety (Azhari et al., 2020). However, student-faculty interaction can help reduce the negative effects of overprotective parenting style and increase their self-efficacy to solve problems independently, which highlights the compensatory effects of student-faculty interaction on emotion regulation.

### Gender differences in parenting styles, student-faculty interaction and emotion regulation

Gender differences in parenting styles may be related to cultural background. Families in western countries are more inclined to define boundaries between parents and children in order for children to achieve independent and autonomous living and to facilitate their emotion regulation. However, socialized values that emphasize obedience, family obligations, and interdependence lead to children relying on their parents for help when problems arise and being more likely to develop negative emotions in Chinese or Asian families (Benito-Gomez et al., 2020). It is important to note that in traditional Chinese culture, boys are often expected to have higher academic and social achievements due to the influence of social gender expectations, and parents’ mentality of “expecting their sons to turn out dragons” intensifies the love and even favoritism of boys (Hannum et al., 2009). In contrast, girls are brought up to be more submissive, gentle, and hardworking, and their parents do not encourage their independence enough (Zhang et al., 2022). Certainly, as people become more educated, contemporary parents are placing more and more emphasis on equality and respectful parenting.

There are inconsistent findings on student-faculty interaction. For instance, Kim and Sax (2009) noted that male students were more likely than female students to volunteer or assist faculty in academic research, and that men had more frequent student-faculty interaction at campus events than women, and that men seemed to prefer active student-faculty interaction in public settings, while women tended to have one-on-one academic interaction. Cohen (2018) indicated that female students were more likely to interact with faculty in frequent academic interaction and focus on getting immediate help. Although the current relevant studies investigated the differences in student-faculty interaction regarding gender (Glazer-Raymo, 2009), but an analysis of gender differences in the types of student-faculty interaction and emotion regulation relationship was so limited.

There are gender differences in emotion regulation, with women likely to experience more negative emotional distress. Although women may use adaptive strategies more than men, this does not help prevent distress; in contrast, women’s use of maladaptive strategies more than men puts them at increased risk for distress (Nolen-Hoeksema and Aldao, 2011). In the unique traditional Chinese cultural context, owing to gender differences in parenting styles, student-faculty interaction and emotion regulation, there are differences in the interrelationships among these three variables.

Based on the above literature review, the study proposes the following hypotheses:

Hypothesis 1 (H1). Here are gender differences in parenting styles, student-faculty interaction and emotion regulation.

Hypothesis 2 (H2). There is a positive relationship between warm parenting style and emotion regulation.

Hypothesis 3 (H3). There is a negative relationship between overprotective parenting style and emotion regulation.

Hypothesis 4 (H4). Student-faculty interaction mediates the relationship between warm parenting style and emotion regulation.

Hypothesis 5 (H5). Student-faculty interaction mediates the relationship between overprotective parenting style and emotion regulation.

## Methodology

### Participants

A total of 4,500 questionnaires were distributed to undergraduate students in Zhejiang Province, mainland China, and 4,462 questionnaires remained after eliminating invalid questionnaires, with a valid return rate of 99.2%. As for the distribution of the sample, in terms of gender, there were 1,108 males (24.8%) and 3,354 females (75.2%). In terms of major types, 2,629 students (58.9%) were in humanities and social sciences; 1,833 students (41.1%) were in science and technology; in terms of grades, 2,179 students (48.8%) were freshman, 1,381 students (31%) were sophomores, 638 students (14.3%) were juniors, and 264 students (5.9%) were seniors.

### Instruments

The following three scales were designed by using a five-point Likert scale, ranging from ‘very non-conforming’ to ‘very conforming’ (on a scale of 1–5), with higher scores indicating better performance or proficiency in this area. The reliability of the scale in this study was also tested by validation factors (Table 1), including topic reliability, component reliability, and convergent validity. Topic reliability is a test of the validity of the topic measures for each dimension, component reliability is the internal consistency of the measures for each construct, and convergent validity is an estimate of the average of the explanatory power of the constructs for the measures. The ideal criteria for the reliability of the scale are Std > 0.6, SMC > 0.4, CR > 0.7 and AVE > 0.5.

**TABLE 1:** Validation factor analysis.

Dimension	Item reliability			Composition Reliability	Convergent Validity
	
	Std	Z	SMC		
Warm parenting style	0.824–0.890	69.502–79.700	0.679–0.793	0.949	0.755
Overprotective parenting styles	0.769–0.901	56.074–67.431	0.591–0.820	0.940	0.724
Academic interaction	0.848–0.921	84.987–103.288	0.719–0.848	0.948	0.783
Social interaction	0.918–0.954	114.798–133.895	0.843–0.910	0.968	0.884
Emotion regulation	0.748–0.839	54.221–62.846	0.560–0.704	0.896	0.633

#### Parenting style scale

Refer to the Family Parenting Style Questionnaire designed by Roo et al. (2022) (S-EMBU). The Chinese version of the Emotionally Warm and Overprotective Family Parenting Style Scale, with 12 items, was revised for the Chinese context. The warm parenting style scale includes six questions such as “having a warm and close feeling with parents.” The overprotective parenting style scale includes six questions such as “interfering with everything I do.” The reliability of the questionnaire is good as the factor loadings for each question item on the Parenting Style Scale are greater than 0.6, the component reliability is 0.949 and 0.940, and the convergent validity is 0.755 and 0.724.

#### Student-faculty interaction scale

Refer to American “National Survey of Student Engagement” (Gordon et al., 2008). The questionnaire has been somewhat revised according to the specific educational situation in China, based on the “space of interaction (distant/face-to-face) and the nature of interaction (directive/functional)” in terms of “student-faculty interaction.” Generating sub-dimensions of student-faculty interaction for undergraduate students: student-faculty academic interaction and social interaction. The measurement of academic student-faculty interaction includes five questions such as “questioning or discussing actively in class” and the measurement of social interaction includes four questions such as “discussing issues of life and values with teacher.” The factor loadings for both academic and social student-faculty interaction were above 0.8, with component reliabilities of 0.948 and 0.968 and convergent validities of 0.783 and 0.884, giving the questionnaire good reliability and validity.

#### Emotion regulation scale

Adapt the OECD-designed framework for measuring emotion regulation based on the Big Five personality theory (De Fruyt et al., 2015). There are 7 items like “I was able to stay calm even in a stressful situation” and so on. The questionnaire had a minimum factor loading of 0.748 for each dimension measured, a compositional reliability of 0.633 and a convergent validity of 0.633, giving the questionnaire good reliability and validity.

### Data analysis

This study used SPSS 23.0 and AMOS 24.0 for data analysis, with the main methods of analysis including reliability analysis, descriptive statistics, correlation analysis, structural equation modeling path analysis and mediating effect tests. One of the tests for mediating effects was to test for mediating effects using a bias-corrected non-parametric percentile Bootstrap method with 1,000 replicate samples to estimate 95% confidence intervals for the mediating variable and to test whether the mediating effect of student-faculty interaction holds.

## Results

### Common method bias

To test for the presence of common method bias, the study used Harman’s one-way test for common method bias, with a factor analysis of all entries, judged by an eigenvalue greater than 1 before rotation. The number of common factors with a characteristic root greater than one was found to be five, and the amount of variance explained by the first of all factors explained by the total variance was 38% less than 40%, indicating that there was no significant common method bias in the data (Podsakoff et al., 2003).

### Descriptive statistics and intercorrelations

Table 2 presents the means and standard deviations of the variables and the Pearson correlation coefficient matrix between the variables. The results show that in terms of student-faculty interaction, the mean value of social student-faculty interaction among undergraduate students is lower than that of academic student-faculty interaction, and it can be assumed that in the Chinese context, undergraduate students prefer academic interaction with teachers and less social interaction outside the classroom. In terms of parenting style, the undergraduate students are better off in terms of warm parenting and the mean value of overprotective parenting is relatively low, suggesting that Chinese parents are more inclined to adopt warm parenting in the family education. The correlation analysis found mostly significant positive relationships between different parenting styles, different types of student-faculty interactions and undergraduate students’ emotion regulation variables, which provided the initial conditions for the subsequent analysis of the structural equation model mediating effects test.

**TABLE 2:** Descriptive statistics and correlation analysis of the variables.

Variables	1	2	3	4	5
1. Warm parenting style	–				
2. Overprotective parenting styles	0.02	–			
3. Academic interaction	0.49**	0.33**	–		
4. Social interaction	0.40**	0.38**	0.83**	–	
5. Emotion regulation	0.60**	0.18**	0.59**	0.52**	–
Mean	4.10	2.87	3.44	3.23	3.81
SD	0.75	0.99	0.83	0.94	0.75

*p < 0.05, **p < 0.01.

Table 3 present the Pearson correlation coefficient matrices between the variables of warm parenting style, overprotective parenting style, academic student-teacher interaction, social student-faculty interaction, and emotion regulation for males and females, respectively. The correlations between the variables differed by gender, with a positive relationship between overprotective and warm parenting styles for males, indicating that for males, parents may adopt a mixture of overprotective and warm parenting styles, but no correlation was shown for females. Second, the correlations between overprotective parenting style and other variables were higher for the male students than for the female students. Moreover, the correlation coefficients between male students’ emotion regulation and academic and social student-faculty interaction were higher, which reflect the fact that male students are more likely to obtain emotional improvement in student-faculty interaction.

**TABLE 3:** Correlation analysis of variables in different gender sample.

Variables	1	2	3	4	5
1. Warm parenting style	–	−0.04*	0.48**	0.38**	0.59**
2. Overprotective parenting styles	0.18**	–	0.27**	0.32**	0.13**
3. Academic interaction	0.53**	0.46**	−	0.82**	0.58**
4. Social interaction	0.44**	0.50**	0.86**	–	0.50**
5. Emotion regulation	0.64**	0.28**	0.61**	0.55**	–

*p < 0.05, **p < 0.01. The correlation matrix for the male sample is below the diagonal, and the correlation matrix for the female sample is above the diagonal.

### Gender difference *t*-test

Significance tests for differences in the main study variables by gender. The results of the study (Table 4) showed that boys had significantly higher levels of emotion regulation than girls (*t* = 6.305, *d* = 0.217). In addition, male families are more likely to adopt an overprotective parenting style than female families in terms of family parenting styles (*t* = 10.842, *d* = 0.376). In terms of student-faculty interaction, boys have more academic and social interaction (*t* = 7.593, *d* = 0.262) than girls (*t* = 8.031, *d* = 0.277) significantly. Hypothesis 1 is verified.

**TABLE 4:** Tests for differences in key variables by gender.

Variables	M ± SD		T	Cohen’s d
	
	Male	Female		
Warm parenting style	4.148 ± 0.764	4.077 ± 0.739	1.734	0.095
Overprotective parenting styles	3.297 ± 0.909	2.980 ± 0.818	10.842***	0.376
Academic interaction	3.605 ± 0.919	3.388 ± 0.793	7.593***	0.262
Social interaction	3.424 ± 1.044	3.164 ± 0.898	8.031***	0.277
Emotion regulation	3.743 ± 0.667	3.605 ± 0.624	6.305***	0.217

***p < 0.001.

### The mediating role of student-faculty interaction in the relationship between parenting style and emotion regulation

**FIGURE 1:**
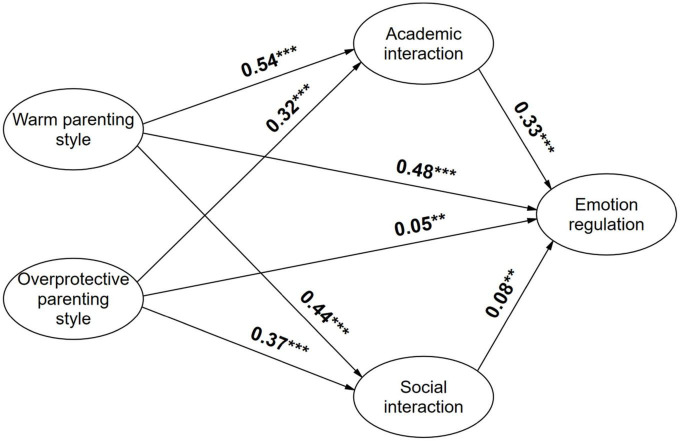
Map of impact path coefficients. *p < 0.05; **p < 0.01; ***p < 0.001.

The relationship between parenting style and undergraduate students’ emotion regulation was examined through structural equation modeling, and maximum likelihood estimation was used to estimate the fit indices of the model. The results show that the various fit indices are X^2^ = 9815.802, df = 998, X^2^/df = 9.835, CFI = 0.918, TLI = 0.918, RMSEA = 0.078. All the model fit indicators passed the test and the model fit was good. As shown in Figure 1 below, warm parenting style has a significant positive impact on undergraduate students’ emotion regulation (β = 0.48, *p*-values < 0.001). Hypothesis 2 is supported. Overprotective parenting and emotion regulation are correlated positively and significantly (β = 0.05, *p*-values < 0.01). Hypothesis 3 is not supported. However, the influence is very weak, as the estimated coefficients are standardized path coefficients, and the comparison shows that the influence of overprotective parenting on the emotion regulation of undergraduate students is only 10% of the effect of warm family parenting.

To test the mediating effect of student-faculty interaction, a bias-corrected non-parametric percentile Bootstrap method was used (Wen et al., 2010). Mediating effect estimates were calculated for each sample and 95% confidence intervals were estimated for the mediating effects (Table 5).

**TABLE 5:** Intermediary effects test.

Path(mediation effect test)	Point Estimate	BootStrap SE	Bootstrapping 95% CI		Mediation effect proportion
			
			Lower	Upper	
**Warm parenting style→Emotion regulation**					
Total effect	0.695	0.014	0.665	0.719	–
Direct effect	0.481	0.020	0.437	0.516	–
Indirect effect(academic interaction)	0.180	0.016	0.151	0.212	25.9%
Indirect effect(social interaction)	0.034	0.011	0.012	0.057	4.9%
**Overprotective parenting style→Emotion regulation**					
Total effect	0.187	0.015	0.157	0.216	–
Direct effect	0.051	0.014	0.023	0.076	–
Indirect effect(academic interaction)	0.107	0.011	0.088	0.130	57.2%
Indirect effect(social interaction)	0.029	0.010	0.011	0.050	15.5%

SE, standard error.

The mediating effects of academic [β = 0.180, SE = 0.016, 95%CI = (0.151, 0.212)] and social [β = 0.034, SE = 0.011, 95%CI = (0.012, 0.057)] student-faculty interaction were significant in the influence of warm parenting style on emotion regulation of undergraduate students, with a total mediating effect of 30.8%. Of these, 25.9% were mediated by academic student-faculty interaction and 4.9% were mediated by social student-faculty interaction.

The mediating effects of academic [β = 0.107, SE = 0.011, 95%CI = (0.088, 0.130)] and social [β = 0.029, SE = 0.010, 95%CI = (0.011, 0.050)] student-faculty interaction were significant in the influence of overprotective parenting style on undergraduate students’ emotion regulation. Total intermediation effect of 72.7%. The proportion of mediated effects was 57.2% for academic student-faculty interaction and 15.5% for social student-faculty interaction.

In conclusion, the mediating effect of both academic and social student-faculty interaction in the relationship between warm-overprotective family parenting style and undergraduate students’ emotion regulation holds, and hypothesis 4 is supported. At the same time, we found that academic student-faculty interaction mediated effects to a much greater extent than social student-faculty interaction, and that student-faculty interaction mediated effects to a greater extent in the relationship between overprotective parenting styles and emotion regulation.

### The gender difference in the mediating role of student-faculty interaction in the relationship between parenting style and emotion regulation

**FIGURE 2:**
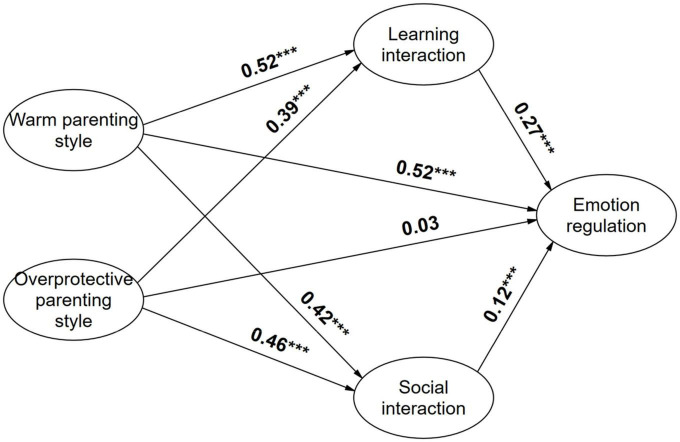
Map of impact path coefficients for male sample. *p < 0.05; **p < 0.01; ***p < 0.001.

**FIGURE 3:**
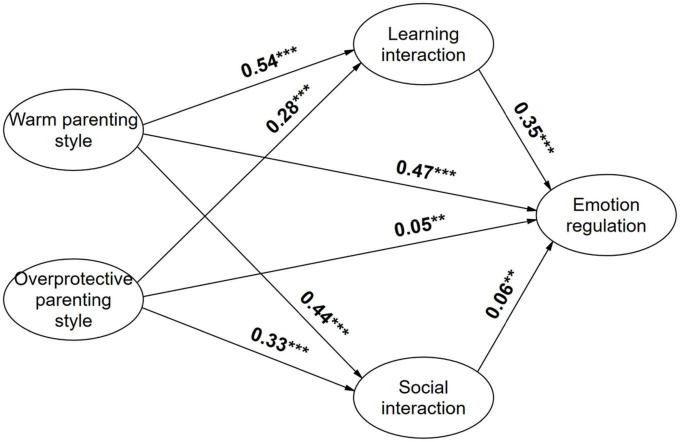
Map of impact path coefficients for female sample. *p < 0.05; **p < 0.01; ***p < 0.001.

The gender differences in the relationship between parenting styles and emotion regulation are examined in Figures 2, 3 below, from which it can be seen that in the path of parenting styles influence on emotion regulation, the coefficients of males in the path of academic interaction are lower than those of female samples, while the coefficients of males in the path of social interaction are higher than those of female samples, indicating that social student-faculty interaction seems to be more beneficial to male students’ emotion regulation, while academic interaction seems to be more favorable to female students’ emotion regulation.

We continued to compare gender difference in the mediating role of student-faculty interaction. As shown in Table 6, the mediating effects of both academic student-faculty interaction and social student-faculty interaction were significant during the influence of warm parenting style on emotion regulation. But there were differences in the influence of men and women, with the proportion of mediating effects of academic student-faculty interaction being lower in the male sample than in the female (19.6% < 27.6%), when the proportion of mediating effects of social student-faculty interaction being higher in the male than in the female students (7.1% > 4.1%). We noticed the same findings during the effect of overprotective parenting style on emotion regulation, with lower mediating effect for academic student-faculty interaction in the male sample than in the female sample (55.5% < 60.2%), but a similarly higher proportion of mediating effect for social student-faculty interaction in the male sample than in the female (29.6% > 12.6%). In summary, the mediating effect of social student-faculty interaction was higher in the male sample than in the female, but the mediating effect of academic student-faculty interaction was lower in the male sample than in the female, and comparing the total effect revealed that the positive association between parenting style and emotion regulation was higher in the male sample than in the female. Hypothesis 5 was supported.

**TABLE 6:** Intermediary effects test.

Path(mediation effect test)		PointEstimate	BootStrapSE	Bootstrapping 95% CI		Mediation effect proportion
				
				Lower	Upper	
**Warm parenting style→Emotion regulation**						
Male	Total effect	0.714	0.026	0.663	0.661	-
	Direct effect	0.523	0.038	0.447	0.593	-
	Indirect effect(academic interaction)	0.140	0.034	0.071	0.203	19.6%
	Indirect effect(social interaction)	0.051	0.025	0.008	0.104	7.1%
Female	Total effect	0.689	0.016	0.656	0.722	-
	Direct effect	0.471	0.022	0.430	0.514	-
	Indirect effect(academic interaction)	0.190	0.017	0.154	0.222	27.6%
	Indirect effect(social interaction)	0.028	0.012	0.006	0.053	4.1%
**Overprotective parenting style→Emotion regulation**						
Male	Total effect	0.189	0.029	0.135	0.250	-
	Direct effect	0.028	0.031	−0.034	0.089	-
	Indirect effect(academic interaction)	0.105	0.026	0.059	0.162	55.5%
	Indirect effect(social interaction)	0.056	0.028	0.007	0.115	29.6%
Female	Total effect	0.166	0.017	0.135	0.201	-
	Direct effect	0.045	0.016	0.018	0.080	-
	Indirect effect(academic interaction)	0.100	0.011	0.079	0.121	60.2%
	Indirect effect(social interaction)	0.021	0.009	0.005	0.042	12.6%

## Discussion

This study examines the influences of different parenting styles on undergraduate students’ emotion regulation and analyses the mediating role of student-faculty interaction. The results show that Chinese parents prefer overprotective parenting styles for male students. Besides, male students have significantly higher level of emotion regulation and student-faculty interaction than female students. In addition, the warm parenting style has a stronger significant positive effect on emotion regulation than the overprotective parenting style. The results of the mediation effect test indicates that the warm-overprotective parenting style indirectly influenced students’ emotion regulation through student-faculty interactions. Interestingly, the level of mediation effect of academic student-faculty interaction is higher than social student-faculty interaction.

### Gender differences in parenting styles, student-faculty interaction, and emotion regulation

In terms of parenting styles, Chinese families tend to be more overprotective of boys than of girls. In Chinese family culture, there is a tendency to over-interfere with boys due to a preference for male ‘inheritance’, especially in cases where there are multiple children, adding to the tendency to be extra protective of boys (Fan and Chen, 2020). Overprotective parenting is the shackle of love. They are full of ‘insecurities’ about their children’s lives, solving problems for them which hinders the development of their children’s independence of thought. By the time they enter the university, men in particular are placed with high educational or career expectations by their parents. Actually, men tend to crave more freedom and independence and need to establish a sense of boundaries in the parent-child relationship to gain a sense of self-efficacy and emotion regulation with independent problem-solving skills (Greene et al., 2018).

As for student-faculty interaction, boys have significantly higher academic and social interaction than girls. To some extent, resource substitution theory (Ross and Mirowsky, 2006), the explanatory power is stronger in the group of boys. After being removed from the excessive parental care and control of their lives, boys come to university with a greater need to develop new social networks. They are more inclined to interact with peer groups and teachers in an open and inclusive manner. In addition, they are more motivated to accumulate social capital in order to reduce even counteract the negative effects of an overprotective upbringing.

As for emotion regulation, boys are significantly more capable of regulating their emotions than girls. This is similar to the findings of Salavera et al. (2022) study. There are some differences in the choice of cognitive emotion regulation strategies between males and females. To be specific, girls often use ruminative thinking that means continuous thinking about loaded emotions. It predicts undesirable emotions such as depression, anxiety and anger. However, males uses more adaptive strategies than females, to the extent that boys are able to regulate their emotions better. In addition, different gender carries different expectations in the socialization process, with women tending to play emotional roles, who are better at perceiving subtle emotional cues and more likely to be disturbed by emotional factors. But men show sensitivity will be suspected of being “unmasculine.” Uniquely, under the influence of traditional Chinese culture, Confucianism encourages individuals to overcome difficulties with resilience, and this ideal personality and sense of responsibility provides relief for boys’ negative emotions in particular (Davis et al., 2012).

### The relationship between parenting style and emotion regulation

Both warm and overprotective parenting styles have a significant positive direct effect on emotion regulation in undergraduate students, and the effect of warm parenting style on emotion regulation is much higher than that of overprotective parenting style.

Persons tend to be more optimistic, loyal and reliable when they are in a harmonious and democratic atmosphere (Pan et al., 2021; Benedetti et al., 2022). Steinberg et al. (1991) pointed out that parenting styles characterized by acceptance, democracy and warmth were the main ways to promote emotion regulation in adolescents, regardless of their social race and socio-economic status. The present study also confirms this finding.

Skinner et al. (2005) suggested that parenting styles characterized by overprotection would hinder the growth of students’ emotion regulation skills. In contrast, this study found that overprotective parenting styles still contributed to emotion regulation. Negative parenting styles may result in students being “psychologically immune,” with some students being forced to ignore or suffer the stress of their parents’ controlling behavior in order to maintain their mental health. Of course, overprotective parenting may also allow students to miss out on numerous opportunities for independent problem solving, resulting in less emotional distress, high levels of self-contentment and good self-evaluation of emotion regulation. However, the data confirms that the effect of overprotective parenting style on emotion regulation is very weak, amounting to only 10% of the effect of warm parenting style. Some studies have found additional negative effects such as anxiety and social fear (Spokas and Heimberg, 2009). It is important to note that Orgilés et al. (2018) suggests that with shifts in family education preferences, particularly in developing countries, parents prefer overprotective parenting styles. This may hinder the development of emotion regulation in children. Therefore, parents need to take a rational look at the impact of parenting styles on their children.

In addition, this study found that the direct effect of parenting style on undergraduate students’ emotion regulation remained significant after the introduction of student-faculty interaction as a mediating variable. It suggested that warm or overprotective parenting style, as a micro-environmental variable in the family, worked partly through student-faculty interaction on undergraduate students’ emotion regulation, but the direct effect was undeniable.

### The mediating role of student-faculty interaction

The mediating role of academic student-faculty interaction in the influence of warm-overprotective parenting style on undergraduate students’ emotion regulation was significant, accounting for 25.9% and 57.2% of the total mediating effect, respectively. Self-determination theory suggests that parenting styles characterized by warmth and support will meet children’s psychological needs, help them internalize their parents’ values and behavioral expectations, and reinforce their willingness to bond with their school teachers (Sinclair et al., 2017). According to resource conservation theory, warm parenting style is a good family resource and academic student-faculty interaction is an important campus resource, both of which provide intellectual support to individuals. It will facilitate the construction and maintenance of internal psychological resources and enhance the accumulation of psychological resources through the “gaining spiral effect,” which is the psychological basis for the development of college students’ emotion regulation (Hobfoll et al., 2018). In particular, undergraduate students who perceive interactive support from teachers through academic interaction could shape good task performance and clear self-perceptions, further exercising their critical and growth mindsets, helping them to gradually work through the negative effects of their family of origin and alleviating the constraints of overprotective parenting styles on emotion regulation (Cuseo, 2018).

Social student-faculty interaction mediated significantly between warm and overprotective parenting style and undergraduate students’ emotion regulation, but the extent of the mediating effect was low, at 4.9% and 15.5%. The weak mediating effect of social student-faculty interaction on family parenting styles on emotion regulation may be due to the fact that student-faculty interaction are more based on academic context. Teachers are under great pressure to teach and do the research, and students have different personality traits. Their time and energy are so limited, which reduces the communication and empathy between them in terms of attitudes and values. These factors reduce the contribution of social student-faculty interaction to students emotion regulation.

The mediating effect of student-faculty interaction on emotion regulation in the warming approach was weaker than in the overprotective parenting approach, with the proportion of the mediating effect of student-faculty interaction being 30.8% in the warming approach and 72.7% in the overprotective parenting approach. On the one hand, the warm family parenting style has more direct influence on emotion regulation. On the other hand, it also indirectly contributes to emotion regulation through student-faculty interaction at school. From this perspective, a warm, understanding and democratic approach to parenting is a more effective way of cultivating emotion regulation skills. Importantly, the effect of overprotective parenting on emotion regulation is low in terms of direct effects, but more indirectly through student-faculty interaction, suggesting that student-faculty interaction in university have a ‘substitution effect’ to compensate for the lack of overprotective parenting in nurturing emotion regulation. Based on resource substitution theory (Ross and Mirowsky, 2006), there is a reciprocal relationship between the impact of different types of resources on an individual’s psychological and emotional well-being. After leaving their origin families, individuals who have been under the negative parental influence for a long time can access social support resources such as peers and teachers at school, all of which help to reduce or even counteract the negative impact of excessive parental intervention on the emotion regulation.

### Gender differences in the mediating role of student-faculty interaction

Based on the analysis of the male-female mediation model, we found that the positive association between parenting styles and emotion regulation was higher in the male sample than in the female sample, and the mediating effect of social student-faculty interaction was higher in the male sample, but the mediating effect of academic student-faculty interaction was lower in the male sample. Sax et al. (2005) held the view that there were differences in the patterns of student-faculty interaction between male and female students. Male students gained more in terms of political participation and social action from their social interactions with teachers than did female students. The positive effect of student-faculty interaction on students’ academic well-being was more pronounced among females, and female students were also more likely to engage in one-on-one academic interaction with teachers (Kim, 2019). Archbell and Coplan (2021) indicated that women exhibited more sensitivity in student-faculty interaction and higher level of social anxiety compared to men. These studies reflect differences in the functions performed by student-faculty interaction that may result from gender differences, with male students being more motivated to engage in social student-faculty interaction, while female students tend to be academic-oriented in their interaction.

## Conclusion

Firstly, male students have significantly higher emotion regulation, overprotective parenting styles and student-faculty interaction scores than female students. Secondly, parenting style has a direct effect on undergraduate students’ emotion regulation, with both warm and overprotective parenting styles having a significant positive effect on emotion regulation. But the influence of overprotective parenting style is very weak. The impact of warm parenting style on emotion regulation relies more on direct influence, while the impact of overprotective parenting style on emotion regulation relies more on the mediating effect of student-faculty interaction. Thirdly, academic student-faculty interaction is the main mediating variable between parenting styles and emotion regulation, with lower level of mediating effect for social student-faculty interaction. In conclusion, our study found that parenting style and student-faculty interaction play an important role in undergraduate students’ emotion regulation, and our findings supported the theoretical guidance of ecosystem theory and emotional development theory in Chinese family and school education contexts, and extend the theory in a more microcosmic way. Finally, we found that male and female students differed in the mechanisms influencing parenting style, student-teacher interactions, and emotion regulation. Parenting style was more positively associated with emotion regulation in the male sample, and male students received more emotion regulation exercises from social student-faculty interaction, while female students promote emotion regulation by academic student-faculty interaction. On the one hand, our findings confirm that undergraduate students’ emotional development is indeed influenced by both family parenting styles and school interaction environment, which is consistent with international research (Saariaho et al., 2018; Efstratopoulou et al., 2022). On the other hand, our survey based on Chinese undergraduate students revealed some novelty findings comparing to international studies, namely, gender differences in the relationship between parenting styles, student-faculty interaction, and emotion regulation might arise from different gender values in China’s unique traditional culture (Zhang, 2006).

## Educational implications

In the context of the Covid-19 pandemic, the mental health of undergraduate students is facing great challenges. It is crucial to guide the development of their emotion regulation skills. Hence, we put forward the following educational recommendations for the positive connection between family parenting styles, student-faculty interaction, and emotion regulation of undergraduate students. First, in order to promote the psycho-emotional well-being of undergraduate students, we should encourage the discovery of more strategies to address undergraduate students’ emotion regulation problems in perspective of school teacher guidance and family education. Parents and teachers should also create a supportive environment that is autonomous, caring, cooperative, and participatory, and establish mutually respectful interpersonal relationships. And we advocate for families to adopt more warm parenting style, and create an atmosphere of trust, encouragement, and supportive parent-child relationships. Second, providing clearer and more detailed guidance to faculty and students on the behavior of interaction especially for students who are in disadvantaged environments. From a gender perspective, we believe that teachers need to consider specific gender differences in interaction, targeting girls who are more introverted and shy, and that girls prefer individual academic interaction, while boys can have group interaction in extra-curricular activities. Besides, suggestions for higher education institutions are to create supportive conditions and climate by motivating faculty to focus on students emotional development. Encouraging students to participate in high-impact educational activities is necessary to promote emotional regulation so as to relieve the psychological stress faced by undergraduate students in the Covid-19 pandemic.

## Limitations

The present study had several limitations that should be recognized. First, this study is a cross-sectional study and no causal inferences can be drawn. Therefore, future research could analyze the short-term or long-term effects of parenting styles on undergraduate students’ emotion regulation from a longitudinal follow-up perspective, and if conditions permit, experimental intervention studies could be conducted to obtain more precise causal judgments. Secondly, this study focuses on the relationship between family parenting style, school student-faculty interaction and undergraduate students’ emotion regulation. In fact, the factors influencing emotion regulation ability are more diverse. For instance, the influence of school peer relationship on college students’ personality also deserves to be studied in depth (Hofmann et al., 2009). It is also necessary to consider how family patterns, such as single-parent families, reconstituted families and other different family patterns, affect the emotion regulation of undergraduate students. Thirdly, how can overprotective parenting styles also positively influence emotional competence to some extent? A combination of qualitative methods such as in-depth interviews may provide new insights into this topic. Finally, teacher-student relationships in Chinese educational contexts tend to follow the ‘authority of the teacher’ and ignore the subjectivity of the student, which may inhibit deep and creative student-faculty communication in a unequal relationship. Interestingly, due to cultural differences, is the effect of social student-faculty interaction on emotion regulation higher than that of academic instruction in the relatively independent, egalitarian and democratic atmosphere of Europe and the United States? Whether there is inter-country variability in our findings in different national contexts is also a theme worth exploring further.

## Data availability statement

The data analyzed in this study is subject to the following licenses/restrictions: The data presented in this study are available on request from the corresponding author. The data are not publicly available due to confidential participant information. Requests to access these datasets should be directed to HY, yaohaoecnu@163.com.

## Ethics statement

Ethical approval was not required in accordance with laws, regulations, and institutional requirements. Completion of the survey implied the participants’ informed consent.

## Author contributions

HY: project administration, writing-original draft, methodology, and data analysis. SC: literature review, conceptualization, and questionnaire survey. XG: supervision, funding acquisition, and editing. All authors contributed equally to the article and approved the submitted version.
